# Sparse spectral graph analysis and its application to gastric cancer drug resistance-specific molecular interplays identification

**DOI:** 10.1371/journal.pone.0305386

**Published:** 2024-07-05

**Authors:** Heewon Park, Satoru Miyano

**Affiliations:** 1 School of Mathematics, Statistics and Data Science, Sungshin Women’s University, Seoul, Republic of Korea; 2 M&D Data Science Center, Tokyo Medical and Dental University, Yushima, Bunkyo-ku, Tokyo, Japan; 3 Human Genome Center, The Institute of Medical Science, The University of Tokyo, Minato-ku, Tokyo, Japan; Universidad de la Republica Uruguay: Facultad de Ingeniería, URUGUAY

## Abstract

Uncovering acquired drug resistance mechanisms has garnered considerable attention as drug resistance leads to treatment failure and death in patients with cancer. Although several bioinformatics studies developed various computational methodologies to uncover the drug resistance mechanisms in cancer chemotherapy, most studies were based on individual or differential gene expression analysis. However the single gene-based analysis is not enough, because perturbations in complex molecular networks are involved in anti-cancer drug resistance mechanisms. The main goal of this study is to reveal crucial molecular interplay that plays key roles in mechanism underlying acquired gastric cancer drug resistance. To uncover the mechanism and molecular characteristics of drug resistance, we propose a novel computational strategy that identified the differentially regulated gene networks. Our method measures dissimilarity of networks based on the eigenvalues of the Laplacian matrix. Especially, our strategy determined the networks’ eigenstructure based on sparse eigen loadings, thus, the only crucial features to describe the graph structure are involved in the eigenanalysis without noise disturbance. We incorporated the network biology knowledge into eigenanalysis based on the network-constrained regularization. Therefore, we can achieve a biologically reliable interpretation of the differentially regulated gene network identification. Monte Carlo simulations show the outstanding performances of the proposed methodology for differentially regulated gene network identification. We applied our strategy to gastric cancer drug-resistant-specific molecular interplays and related markers. The identified drug resistance markers are verified through the literature. Our results suggest that the suppression and/or induction of COL4A1, PXDN and TGFBI and their molecular interplays enriched in the Extracellular-related pathways may provide crucial clues to enhance the chemosensitivity of gastric cancer. The developed strategy will be a useful tool to identify phenotype-specific molecular characteristics that can provide essential clues to uncover the complex cancer mechanism.

## Introduction

Gastric cancer is a leading cause of cancer-related deaths worldwide. Chemotherapy is the standard gastric cancer treatment. However, chemotherapy is not always effective because cancer cells acquire resistance to targeted drugs. Several studies have been conducted to uncover drug resistance mechanisms in cancer chemotherapy in various research fields, including medicine, health sciences, and pharmaceutical sciences. Although many bioinformatics studies attempted to uncover the drug resistance mechanism and related molecular characteristics, most studies were based on individual or differential gene expression (DEG) analysis [1, 2]. However, the complex mechanisms underlying drug resistance in cancer cells cannot be understood by perturbation in a single gene because abnormalities in complex molecular networks cause the mechanisms involved in disease. Therefore, the single gene-based and DEG analysis is insufficient to uncover drug resistance mechanisms and molecular characteristics in cancer cells.

The main aim of this study is to disclose the crucial molecular interplay that is instrumental in the mechanism driving acquired drug resistance in gastric cancer. In order to effectively identify the crucial molecular interplay, we consider drug resistance-specific molecular characteristics identification based on differential gene network (ssDGN) analysis. Recently, several methods have been proposed to differential gene network analysis by comparing graph spectra [1, 3, 4]. The studies identified differential networks based on eigenvalues of adjacency, Laplacian, and normalized Laplacian matrices. However, the eigenanalysis in these studies is based on fully dense loadings and thus suffers from the following drawbacks: *“difficulty in interpretation of the eigenanalysis results, because eigenvalues are estimated as linear combinations of all variables”* and *“the results may be disturbed by noise features included in datasets”*. In order to effectively perform for differentially regulated gene network identification, we develop a novel computational strategy for sparse eigenanalysis of graph spectra called Net-sEVD. Our strategy incorporates sparsity into eigen loading estimation based on *L*_1_-type regulari zation. Thus, only crucial features to describe the graph structure are involved in the eigenanalysis. Furthermore, the sparse loadings lead to effective interpretation of the eigenanalysis results. To achieve a biologically reliable results, we also incorporated knowledge of network biology that *“the genes linked in the networks may have similar biological functions”* and *“the hub genes linked with many genes are crucial markers to understand biological mechanisms”* into the eigen loading estimation [5]. Consequently, our strategy encourages coefficient similarity of the linked genes in the eigenloadings estimation, which leads to the simultaneous selection of the linked genes. Our analysis imposes a relatively small penalty on the hub genes. Thus, hub genes and their neighboring genes have relatively large coefficients and are easily selected. In summary, the spectral graph analysis is based on crucial sub-networks consisting of the hub genes and their neighboring genes. Therefore, we can perform a biologically reliable interpretation of the spectral graph analysis results.

Monte Carlo simulations were conducted to demonstrate the proposed method’s effectiveness. The ssDGN method showed an outstanding performance in identifying differentially regulated gene networks in various scenarios of gene network structures. We applied ssDGN to identify differentially regulated gene networks between gastric cancer-drug-sensitive and -resistant cell lines based on the Sanger Genomics of Drug Sensitivity in Cancer (GDSC) dataset. We then identified gastric cancer drug resistance-specific molecular interplays and related markers. Most of the identified drug resistance markers have strong evidence as biomarkers for gastric cancer and its chemotherapy. Our results with Gene Ontology (GO) term pathway analysis uncovered that “extracellular matrix” (ECM)-related pathways are involved in drug resistance mechanisms, and the COL4A1, PXDN, and TGFBI genes act as drug resistance markers. We suggest that suppression and/or induction of COL4A1, PXDN and TGFBI and their molecular interplays enriched in the extracellular-related pathways may provide crucial clues to enhance gastric cancer’s chemosensitivity.

The remainder of this paper is organized as follows: The methods section introduces the proposed method for sparse spectral graph analysis and ssDGN identification. We discuss simulation studies in the Monte Carlo simulations section. Finally, we describe the results of identifying gastric cancer drug resistance-specific gene networks. Conclusions are provided in the Discussion section.

## Methods

We considered a gene regulatory network that is represented by a weighted undirected graph *G* = (*V*, *E*, *W*), where *V* = {1, …, *p*} is the set of vertices corresponding to *p* genes, *E* ∈ *V* × *V* (i.e., *E* = (*i* ∼ *j*)) is the edge set, and *W* = (*w*_*ij*_), (*i*, *j*) ∈ *E* is the edge weight. The adjacency matrix *A*(*G*) is defined as

$$\begin{array}{c} A(G)=\{\begin{array}{cc} w_{ij} & \text{if}\;i∼j,\\ 0 & \text{otherwise}. \end{array} \end{array}$$ (1)

The degree of a vertex *i* is defined as *d*_*i*_ = ∑_*j*∼*i*_ *w*_*ij*_, and the degree matrix ***D*** is given as the following diagonal matrix

$$\begin{array}{c} D_{ij}=\{\begin{array}{cc} d_{i} & \text{if}\;i=j,\\ 0 & \text{if}\;i\neq j. \end{array} \end{array}$$ (2)

The Laplacian matrix is given as ***L***_0_ = ***D*** − *A*(*G*) and the normalized Laplacian matrix ***L*** = ***D***^−1/2^***L***_0_***D***^−1/2^ is defined as [3, 5],

$$\begin{array}{c} L=l_{ij}=\{\begin{array}{cc} 1-\frac{w_{ij}}{d_{i}} & \text{if}\;i=j\;\text{and}\;d_{i}\neq 0,\\ -\frac{w_{ij}}{\sqrt{d_{i}d_{j}}} & \text{if}\;(i,j)\in E,\\ 0 & \text{otherwise}. \end{array} \end{array}$$ (3)

The gene regulatory network can be described by *A*(*G*), ***L***_0_ and ***L***.

### Existing method to measure similarity of graph

#### Preliminaries

In this section, we discuss the similar matrix and their eigenvalues. The similar matrix is defined as follows (Definition 4.1 of [6]),

**Definition 1** *The p* × *p matrices* ***M***^*Y*^ *and* ***M***^*N*^ *are said to be similar matrices if a nonsingular matrix* **V** *exists, such that*

$$\begin{array}{c} M^{Y}=VM^{N}V^{-1}. \end{array}$$ (4)

We then consider the following property for the *p* × *p* matrix (Theorem 3.2 (d) of [6]).

**Theorem 1** *Let* ***M***^*Y*^ *be an p* × *p matrix. Then, the eigenvalues of* ***VM***^*Y*^***V***^−1^ *are the same as the eigenvalues of* ***M***^*Y*^, *if* ***V*** *is a nonsigular p* × *p matrix*.

The definition of similar matrix and theorem imply that similar matrices ***M***^*Y*^ and ***M***^*N*^ have identical eigenvalues.

#### Graph similarity measure based on Laplacian matrices

The eigenanalysis has been used to describe network structures, and many methods have been developed to measure the networks’ similarity/dissimilarity based on the eigenvalues of adjacency, Laplacian-, and normalized Laplacian matrices [1, 3, 7]. The weighted graphs *G*^*Y*^ and *G*^*N*^ for phenotypes Y and N are represented by *p* × *p* matrices ***M***^*Y*^ and ***M***^*N*^ (e.g., precision matrix, Laplacian matrix, adjacency matrix, etc.), respectively. Suppose ***M***^*Y*^ and ***M***^*N*^ are similar matrices. It follows from Theorem 1 that the matrices ***M***^*Y*^ and ***M***^*N*^ have identical eigenvalues We suppose rank(*A*(*G*^*Y*^)) = rank(*A*(*G*^*Y*^)) = *q* ≤ *p*. Wills and Meyer [3] introduced the following spectral distance to measure the dissimilarity of two graphs

$$\begin{array}{c} d\left(G^{Y},G^{N}\right)=\sqrt{\sum_{r=1}^{q}{\left(\lambda {\left(A(G^{Y})\right)}_{r}-\lambda {\left(A(G^{N})\right)}_{r}\right)}^{2}}, \end{array}$$ (5)

where *q* is the number of eigenvalues for best rank approximation of *A*(*G*^*Y*^) and *A*(*G*^*N*^), and λ(*A*(*G*^*Y*^))_*r*_ and λ(*A*(*G*^*N*^))_*r*_ are *r*^*th*^ eigenvalues of adjacency matrices of *A*(*G*^*Y*^) and *A*(*G*^*N*^), respectively.

Although various graph spectra-based strategies have been proposed to measure network similarity, the existing methods are based on fully dense loadings. However, the fully dense loadings lead to the following drawbacks

- The eigenvalues are estimated as linear combinations of all variables, leading to difficulty interpreting the eigenanalysis results.
- The eigenanalysis can be disturbed by noisy features included in datasets.

To settle on the issues, we developed a novel strategy for sparse eigenanalysis to capture gene network structure.

### Network constrained sparse spectral graph analysis of the graph laplacian matrices

The spectral (eigenvalue) decomposition of the normalized Laplacian matrix ***L*** with rank *q* is defined as

$$\begin{array}{c} L=V\Lambda V^{T}=\sum_{r=1}^{q}\lambda_{r}v_{r}v_{r}^{T}\\ \text{subject}\;\text{to}\;V^{T}V=I_{q}, \end{array}$$ (6)

where ***V*** = [***v***_1_, ***v***_2_, …, ***v***_*q*_] and **Λ** = diag(λ_1_, λ_2_, …, λ_*q*_) with non-negative eigenvalues λ_1_ ≥ λ_2_ ≥ … ≥ λ_*q*_ ≥ 0. The optimization problem for estimating eigen vectors ***V*** in (5) can also be interpreted by the following principal component analysis,

$$\begin{array}{c} {\hat{\alpha }}_{r}=\underset{\alpha }{\text{arg}\;\text{max}}\;\alpha^{T}\hat{\Sigma }\alpha ,\\ \text{subject}\;\text{to}\;\alpha_{r}^{T}\alpha_{r}=1\;\text{and}\;\alpha_{r}^{T}\alpha_{k}=0\;\text{for}\;\text{all}\;r\neq k, \end{array}$$ (7)

where $\hat{\Sigma }=L^{T}L$.

Various methodologies have been developed to estimate sparse loadings. Zou et al. [8] proposed the following sparse PCA

$$\begin{array}{c} \;\left(\hat{A},\hat{B}\right)=\underset{A,B}{\text{arg}\;\text{min}}\{||L-LBA^{T}{\left|\right|}_{F}^{2}+\gamma_{1}\sum_{r=1}^{q}\|\beta_{r}{\|}_{1}+\gamma_{2}\sum_{r=1}^{q}\|\beta_{r}{\|}_{2}^{2}\},\\ \text{subject}\;\text{to}\;A^{T}A=I_{q}, \end{array}$$ (8)

where *γ*_1_, *γ*_2_ > 0 are regularization parameters for ***β***_*r*_, *r* = 1, …, *q*, ***A*** = [***α***_1_, ***α***_2_, …, ***α***_*q*_], ***B*** = [***β***_1_, ***β***_2_, …, ***β***_*q*_]. Guo et al. [9] developed the novel sparse PCA based on fused loadings,

$$\begin{array}{c} \;\left(\hat{A},\hat{B}\right)=\underset{A,B}{\text{arg}\;\text{min}}\{||L-LBA^{T}{\left|\right|}_{F}^{2}+\gamma_{1}\sum_{r=1}^{q}\|\beta_{r}{\|}_{1}+\gamma_{2}\sum_{r=1}^{q}\sum_{s∼t}|\rho_{s,t}\left|\right\|\beta_{s,r}-\text{sign}\left(\rho_{s,t}\right)\beta_{t,r}{\|}_{1}\},\\ \text{subject}\;\text{to}\;A^{T}A=I_{q}, \end{array}$$ (9)

where *ρ*_*s*,*t*_ is the sample correlation between *s*^*th*^ and *t*^*th*^ features in ***L*** and sign(⋅) is the sign function. Croux et al. [10] proposed a robust version of the sparse PCA based on the strong measure of variance. A methodology for sparse loading estimation was developed by incorporating sparsity into singular value decomposition [11]. However, the existing methods were developed purely from mathematical and algorithmic points without considering biological knowledge. Thus, biological interpretation of the results cannot be effectively performed.

To effectively and interpretably perform differentially regulated gene network identification, we proposed a novel strategy for sparse eigenanalysis of the normalized Laplacian matrix ***L***, called Net-sEVD, based on network-constrained regularization [5, 12–14],

$$\begin{array}{cc} \left(\hat{A},\hat{B}\right) & =\underset{A,B}{\text{arg}\;\text{min}}\{||L-LBA^{T}{\left|\right|}_{F}^{2}+\gamma_{1}\sum_{r=1}^{q}\|\beta_{r}{\|}_{1}+\gamma_{2}\sum_{r=1}^{q}\sum_{s∼t}(\frac{\beta_{r,s}}{\sqrt{d_{s}}}-\frac{\beta_{r,t}}{\sqrt{d_{t}}}{)}^{2}w_{st}\}\\ & =\underset{A,B}{\text{arg}\;\text{min}}\{||L-LBA^{T}{\left|\right|}_{F}^{2}+\gamma_{1}\sum_{r=1}^{q}\|\beta_{r}{\|}_{1}+\gamma_{2}\sum_{r=1}^{q}\beta_{r}^{T}L\beta_{r}\},\\ & \text{subject}\;\text{to}\;A^{T}A=I_{q}, \end{array}$$ (10)

where *s* ∼ *t* indicates the set of gene pairs which are connected to *s* in the gene network. In our strategy, the computed Laplacian matrix ***L*** in (3) is used to input of the optimization problem in (10) and the eigenloadings ***B*** = [***β***_1_, ***β***_2_, …, ***β***_*q*_] are regularized by the normalized Laplacian matrix ***L***. The second term of the objective function of our strategy is a quadratic Laplacian penalty, called a Dirichlet energy, that is used for quantifying the smoothness of the graph, and leads to identify important features by measuring their smoothness on the graph structure [15, 16] In our strategy, the quadratic Laplacian penalty used to induce a smooth solution of the parameters ***β***_*r*_ between neighboring vertices over the gene network. Furthermore, the scaling of the coefficients *β*_*r*,*s*_ respect to the degree (i.e., $\sqrt{d_{s}}$) allows the hub gene with more connections to have larger coefficients *β*_*r*,*s*_. The use of the quadratic Laplacian penalty enables us to incorporate the following knowledge of network biology into the eigen loading estimation [5].

- *The genes linked in the networks may have similar biological functions*
  Using the network-constrained penalty, we encouraged the linked genes’ similarity of coefficients in the eigenloadings estimation. The penalty term enabled us to locally smooth the network and encourage the simultaneous selection of related features, the large value of *w*_*st*_ (i.e., strong edge between *s*^*th*^ gene and *t*^*th*^ gene) lead to a large amount of penalty to difference of *β*_*r*,*s*_ and *β*_*r*,*t*_. It implies that the last term of our method leads to connected genes with strong edges have similar *β*_*r*,*s*_ and *β*_*r*,*t*_ (*r* = 1, …, *q*) in loading matrix estimation.
- *The hub genes linked with several genes play critical roles in gene regulation and biological processes* [17].
  The hub genes are crucial markers for understanding biological mechanisms. Our strategy imposed a relatively small penalty on the hub genes by re-scaling the coefficients with the square root of the degrees *d*_*s*_. Thus, the hub genes and their neighboring genes have relatively large coefficients and are easily selected.

In short, our strategy’s eigenvalue estimation is based on crucial sub-networks consisting of the hub genes and their neighboring genes, which leads to a biologically reliable interpretation of the spectral graph analysis results and crucial markers identification.

#### Algorithm of Net-sEVD

To implement Net-sEVD, we adapted the iterative algorithm of the sparse PCA [8]

**Step 1**. Initialize $\hat{A}$ at $\hat{V}$ by setting it to the eigenvalue decomposition of ***L***.
**Step 2**. For given $\hat{A}$, the sparse eigenloadings are estimated by solving the following optimization problem for *r* = 1, …, *q*,
  $$\begin{array}{c} {\hat{\beta }}_{r}=\underset{\beta_{r}}{\text{arg}\;\text{min}}\left\{\right\|z_{r}^{*}-L\beta_{r}{\|}^{2}+\gamma_{1}\|\beta_{r}{\|}_{1}+\gamma_{2}\beta_{r}^{T}L\beta_{r}\}, \end{array}$$
  where $z_{r}^{*}=L\alpha_{r}$ Update $\hat{B}=\left[{\hat{\beta }}_{1},{\hat{\beta }}_{2},\ldots ,{\hat{\beta }}_{q}\right]$.
**Step 3** For fixed $\hat{B}$, compute SVD of $L^{T}L\hat{B}=V\Lambda V^{T}$ [8]. Update $\hat{A}=VV^{T}$ and $Z=L\hat{A}$.
**Step 4** Repeat Steps 2–3 until convergence.
**Step 5** Normalize ${\hat{v}}_{r}$ as ${\hat{\beta }}_{r}/\left\|{\hat{\beta }}_{r}\right\|$ for *r* = 1, 2, …, *q*, and update ***V*** = [***v***_1_, …, ***v***_*q*_].
**Step 6** Compute **Λ**^*s*^ = ***V***^*T*^***LV***

### Differential gene regulatory networks identification based on Net-sEVD: ssDGN

Suppose the normalized Laplacian matrices *L*^*Y*^ and *L*^*N*^ that describe the estimated gene networks for *p* genes, where the networks are estimated by expression levels of phenotypes Y and N. By using the Net-sEVD, we first estimate eigenvalues λ^*s*^(*L*^*Y*^) and λ^*s*^(*L*^*N*^) of *p* × *p* matrices *L*^*Y*^ and *L*^*N*^, respectively. We then measured the dissimilarity of gene regulatory networks based on the following distance *d*^*s*^(*G*^*Y*^, *G*^*N*^) of eigenvalues in line with [3]

$$\begin{array}{c} d^{s}\left(G^{Y},G^{N}\right)=\sum_{r=1}^{q}{\left\{\lambda^{s}{\left(L^{Y}\right)}_{r}-\lambda^{s}{\left(L^{N}\right)}_{r}\right\}}^{2}, \end{array}$$ (11)

where *L*^*Y*^ and *L*^*N*^ are the normalized Laplacian matrices of the graphs *G*^*Y*^ and *G*^*N*^, and λ^*s*^(⋅)_*r*_ is the *r*^*th*^ eigenvalue of the normalized Laplacian matrix.

To identify differentially regulated gene networks, we considered the permutation p-value of *d*^*s*^(*G*^*Y*^, *G*^*N*^). We draw permutation cell lines of phenotypes Y and N, respectively, and then estimate the permutation gene networks of *p* genes for the phenotypes Y and N, i.e., *G*^*Y*^_*pm*_ and *G*^*N*^_*pm*_. The normalized Laplacian matrices $L_{pm}^{Y}$ and $L_{pm}^{N}$ are computed. The eigenvalues of the permutation matrices are estimated as $\lambda^{s}\left(L_{pm}^{Y}\right)$ and $\lambda^{s}\left(L_{pm}^{N}\right)$. We then computed the permutation p-value of the distance as follows,

$$\begin{array}{c} \text{p}.\text{value}=\frac{\sum_{pm=1}^{T}I\left(d^{s}\left(G^{Y},G^{N}\right)\leq d^{s}\left({G^{Y}}_{pm},{G^{N}}_{pm}\right)\right)}{T}, \end{array}$$ (12)

where *T* is the number of permutations, ***I***(⋅) is an indicator function, *d*^*s*^(*G*^*Y*^_*pm*_, *G*^*N*^_*pm*_) is the distance between eigenvalues of the normalized Laplacian matrices for permutation gene networks *G*^*Y*^_*pm*_ and *G*^*N*^_*pm*_, and *T* is several permutations. The small permutation p-value indicates that networks *G*^*Y*^ and *G*^*N*^ show significantly different gene regulatory structures between two phenotypes, Y and N. The differentially regulated gene network identification was performed by the permutation p-value with significance level *α*.

### Regularization parameter selection

The results of the proposed Net-sEVD heavily relies on the regularization parameters *γ*_1_ and *γ*_2_. Let $A^{\gamma_{1},\gamma_{2}}=\left[\alpha_{1}^{\gamma_{1},\gamma_{2}},\ldots ,\alpha_{q}^{\gamma_{1},\gamma_{2}}\right]$ and $B^{\gamma_{1},\gamma_{2}}=\left[\beta_{1}^{\gamma_{1},\gamma_{2}},\ldots ,\beta_{q}^{\gamma_{1},\gamma_{2}}\right]$ be the estimates of ***A*** and ***B***. We consider the following criterion to select the tuning parameters,

$$\begin{array}{c} C_{\gamma_{1},\gamma_{2}}=||L-LB^{\gamma_{1},\gamma_{2}}{\left(A^{\gamma_{1},\gamma_{2}}\right)}^{T}{\left|\right|}_{F}^{2}. \end{array}$$ (13)

Then, we select the regularization parameters that minimize (13).

## Monte Carlo simulations

We illustrated the performance of the proposed ssDGN based on Monte Carlo simulations. We supposed two phenotypes, A and B, with sample sizes *n*_*A*_ = *n*_*B*_ = 100. We considered five common and five A-specific sub-networks, where each sub-network comprises ten genes.

**Fig 1:**
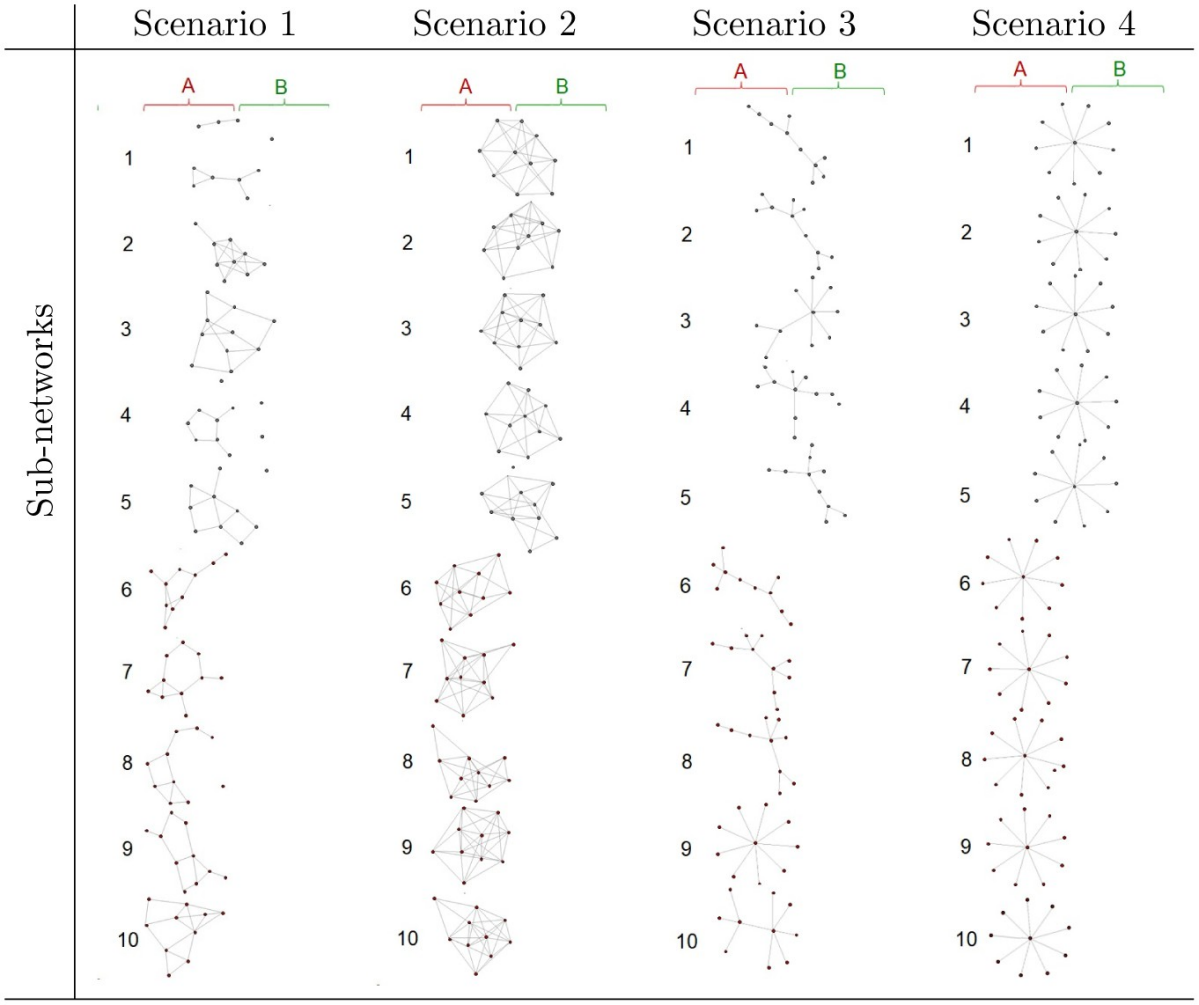
Sub-network structures of Monte Carlo simulations.

In scenario 1, we generated five precision matrices for each common sub-network (i.e., $\Omega_{c}^{nw}$, *nw* = 1,.., 5) and A-specific networks (i.e., $\Omega_{A}^{nw}$, *nw* = 1,.., 5) from “random” graph structure using *huge.generator* an R package Huge previously described [18]. The expression levels of each of the ten genes in every five common networks were generated from $N(0_{10},{\left(\Omega_{c}^{nw}\right)}^{-1})$, where the number of cell lines is *n* = 200. For the A-specific networks, the expression levels ***X***^*A*^ of each of the ten genes comprising each sub-network are generated from $N(0_{10},{\left(\Omega_{A}^{nw}\right)}^{-1})$, where the sample size of phenotype A is *n*_*A*_ = 100. We then generated the expression levels ***X***^*B*^ of phenotype B from *N*(**0**_50_, **Σ**_50_), where **Σ**_50_ is the diagonal matrix whose diagonal is *σ*^2^. Scenarios 2, 3, and 4 are similar to scenario 1 except that the precision matrices $\Omega_{c}^{nw}$ for *nw* = 1,.., 5 and $\Omega_{A}^{nw}$ for *nw* = 1, …, 5 have “cluster,” “scale-free,” and “hub” graph structures for scenarios 2, 3, and 4, respectively. We use significance level *α* = 0.05 and extract the subnetwork corresponding p.value <0.05. Fig 1 shows the graph structures of the common sub-networks and A-specific sub-networks.

In the differentially regulated gene network identification, we first estimate gene regulatory networks *G*^*A*^ and *G*^*B*^ based on the generated expression levels of genes for phenotypes A and B (***X***^*A*^ and ***X***^*B*^), respectively. In the simulation study, we compute the correlation matrices of expression levels of genes ***X***^*A*^ and ***X***^*B*^ to describe gene network (i.e., correlation network) for phenotypes A and B.

$$\begin{array}{c} \rho_{ij}^{A}=\frac{Cov(x_{i}^{A},x_{j}^{A})}{\sigma_{x_{i}^{A}}\sigma_{x_{j}^{A}}}\;\text{and}\;\rho_{ij}^{B}=\frac{Cov(x_{i}^{B},x_{j}^{B})}{\sigma_{x_{i}^{B}}\sigma_{x_{j}^{B}}}, \end{array}$$ (14)

where *Cov*(***x***_*i*_, ***x***_*j*_) is a covariance between expression levels of the *i*^*th*^ and *j*^*th*^ genes, and $\sigma_{x_{i}}$ is a standard deviation of the expression levels of the *i*^*th*^ genes. The absolute values of correlation coefficients are used as edge weights ***W***^*A*^ and ***W***^*B*^,

$$\begin{array}{c} W^{A}=|\rho_{ij}^{A}|\;\text{and}\;W^{B}=\left|\rho_{ij}^{B}\right|. \end{array}$$ (15)

We then compute the normalized Laplacian matrices *L*^*A*^ and *L*^*B*^ in (3). By using the proposed Net-sEVD, we compute eigenvalues of the normalized Laplacian matrices *L*^*A*^ and *L*^*B*^ and then extract phenotype specific sub-network (i.e., the subject of the proposed method Net-sEVD are the normalized Laplacian matrices *L*^*A*^ and *L*^*B*^). We evaluated ssDGN compared with similarity measures based on eigenvalues estimated by fully dense loading (i.e., ordinary EVD: oEVD), sparse loadings (sPCA) [8], sparse loadings (sPCA) without *L*_2_ norm (sPCAwoL2), fused sparse loadings (sfPCA) [9] and sign adjusted quadratic Laplacian penalty $\beta_{r}^{T}L^{s}\beta_{r}$ (ssDGNs), where ***L***^*s*^ = ***S***^*T*^***LS*** with $S=\text{diag}\{\text{sgn}\left({\hat{\beta }}_{r,1}\right),\text{sgn}\left({\hat{\beta }}_{r,2},\ldots ,\text{sgn}\left({\hat{\beta }}_{r,p}\right)\right\}$. The permutation p-values were computed based on 500 permutation samples, and the simulations were repeated 50 times. To evaluate the performance of differential gene network identification, we compare accuracy, F-measure, true negative rate (TNR), recall, and precision. Tables 1–3 show the average of the results in 50 replications for *σ*^2^ = 1, 3 and 5, respectively, where the bold values indicate the best performance among the methods. As shown in Tables 1–3, the proposed ssDGN effectively identified differential regulatory networks for scenarios 1, 3 and 4 in overall. The methods showed similar performance in the viewpoint of Recall (i.e., true positive rate), while our strategy outperformed true negative identification given in the column “TNR”. It can be seen through Tables 1–3 that the proposed ssDGN provides effective performance for differentially regulated gene network identification, overall. We expected that our strategy will be a useful tool to identify phenotype-specific molecular inteplays that can decribed crucial characteristics of cancer mechanism.

**Table 1:** Simulation results: Differential gene network identification *σ*^2^ = 1.

	Methods	Scn.1	Scn.2	Scn.3	Scn.4
Accuracy	ssDGN	**0.984**	0.976	**0.978**	0.978
		(0.037)	(0.048)	(0.046)	(0.042)
	ssDGNs	0.982	0.976	0.972	0.974
		(0.044)	(0.048)	(0.061)	(0.044)
	sfPCA	0.978	0.984	0.976	0.972
		(0.062)	(0.042)	(0.056)	(0.045)
	sPCA	0.978	0.984	0.976	0.974
		(0.051)	(0.042)	(0.056)	(0.049)
	sPCA(woL2)	0.970	0.976	0.972	**0.982**
		(0.046)	(0.052)	(0.045)	(0.039)
	oEVD	0.976	**0.986**	0.974	0.972
		(0.056)	(0.040)	(0.056)	(0.045)
F.measure	ssDGN	**0.985**	0.978	**0.980**	0.980
		(0.034)	(0.042)	(0.041)	(0.038)
	ssDGNs	0.984	0.978	0.975	0.976
		(0.039)	(0.042)	(0.051)	(0.040)
	sfPCA	0.981	0.986	0.979	0.975
		(0.050)	(0.037)	(0.046)	(0.041)
	sPCA	0.981	0.986	0.979	0.977
		(0.044)	(0.037)	(0.046)	(0.043)
	sPCA(woL2)	0.973	0.979	0.974	**0.984**
		(0.042)	(0.045)	(0.043)	(0.035)
	oEVD	0.979	**0.988**	0.977	0.975
		(0.048)	(0.035)	(0.047)	(0.041)
TNR	ssDGN	**0.968**	0.952	**0.956**	0.956
		(0.074)	(0.095)	(0.093)	(0.084)
	ssDGNs	0.964	0.952	0.948	0.948
		(0.088)	(0.095)	(0.120)	(0.089)
	sfPCA	0.956	0.968	0.952	0.944
		(0.123)	(0.084)	(0.111)	(0.091)
	sPCA	0.956	0.968	0.952	0.948
		(0.101)	(0.084)	(0.111)	(0.097)
	sPCA(woL2)	0.940	0.952	0.952	**0.964**
		(0.093)	(0.103)	(0.086)	(0.078)
	oEVD	0.952	**0.972**	0.948	0.944
		(0.111)	(0.081)	(0.113)	(0.091)
Recall	ssDGN	1.000	1.000	1.000	1.000
		(0.000)	(0.000)	(0.000)	(0.000)
	ssDGNs	1.000	1.000	0.996	1.000
		(0.000)	(0.000)	(0.028)	(0.000)
	sfPCA	1.000	1.000	1.000	1.000
		(0.000)	(0.000)	(0.000)	(0.000)
	sPCA	1.000	1.000	1.000	1.000
		(0.000)	(0.000)	(0.000)	(0.000)
	sPCA(woL2)	1.000	1.000	0.992	1.000
		(0.000)	(0.000)	(0.040)	(0.000)
	oEVD	1.000	1.000	1.000	1.000
		(0.000)	(0.000)	(0.000)	(0.000)
Precision	ssDGN	**0.973**	0.961	**0.964**	0.963
		(0.062)	(0.076)	(0.074)	(0.070)
	ssDGNs	0.971	0.961	0.960	0.957
		(0.069)	(0.076)	(0.086)	(0.074)
	sfPCA	0.968	0.974	0.963	0.953
		(0.086)	(0.066)	(0.081)	(0.076)
	sPCA	0.965	0.974	0.963	0.958
		(0.078)	(0.066)	(0.081)	(0.078)
	sPCA(woL2)	0.950	0.962	0.960	**0.970**
		(0.077)	(0.080)	(0.072)	(0.065)
	oEVD	0.963	**0.978**	0.959	0.953
		(0.084)	(0.063)	(0.083)	(0.076)

**Table 2:** Simulation results: Differential gene network identification *σ*^2^ = 3.

	Methods	Scn.1	Scn.2	Scn.3	Scn.4
Accuracy	ssDGN	0.966	0.976	**0.978**	**0.978**
		(0.059)	(0.048)	(0.046)	(0.046)
	ssDGNs	0.974	0.970	0.974	0.974
		(0.049)	(0.054)	(0.049)	(0.056)
	sfPCA	0.976	0.972	0.974	0.976
		(0.048)	(0.054)	(0.049)	(0.052)
	sPCA	**0.978**	0.974	0.974	**0.978**
		(0.046)	(0.053)	(0.053)	(0.046)
	sPCA(woL2)	0.966	**0.980**	0.976	0.972
		(0.063)	(0.040)	(0.048)	(0.057)
	oEVD	0.974	0.970	**0.978**	**0.978**
		(0.049)	(0.054)	(0.051)	(0.051)
F.measure	ssDGN	0.970	0.978	0.980	0.980
		(0.051)	(0.042)	(0.041)	(0.041)
	ssDGNs	0.977	0.973	0.977	0.977
		(0.043)	(0.048)	(0.043)	(0.049)
	sfPCA	0.978	0.975	0.977	0.979
		(0.042)	(0.047)	(0.043)	(0.045)
	sPCA	**0.980**	0.977	0.977	**0.980**
		(0.041)	(0.046)	(0.046)	(0.041)
	sPCA(woL2)	0.970	**0.982**	0.978	0.975
		(0.054)	(0.037)	(0.042)	(0.049)
	oEVD	0.977	0.973	**0.981**	**0.981**
		(0.043)	(0.048)	(0.044)	(0.044)
TNR	ssDGN	0.932	**0.952**	**0.956**	**0.956**
		(0.119)	(0.095)	(0.093)	(0.093)
	ssDGNs	0.948	0.940	0.948	0.948
		(0.097)	(0.109)	(0.097)	(0.113)
	sfPCA	0.952	0.944	0.948	0.952
		(0.095)	(0.107)	(0.097)	(0.103)
	sPCA	**0.956**	0.948	0.948	**0.956**
		(0.093)	(0.105)	(0.105)	(0.093)
	sPCA(woL2)	0.932	0.960	0.952	0.944
		(0.125)	(0.081)	(0.095)	(0.115)
	oEVD	0.948	0.940	**0.956**	**0.956**
		(0.097)	(0.109)	(0.101)	(0.101)
Recall	ssDGN	1.000	1.000	1.000	1.000
		(0.000)	(0.000)	(0.000)	(0.000)
	ssDGNs	1.000	1.000	1.000	1.000
		(0.000)	(0.000)	(0.000)	(0.000)
	sfPCA	1.000	1.000	1.000	1.000
		(0.000)	(0.000)	(0.000)	(0.000)
	sPCA	1.000	1.000	1.000	1.000
		(0.000)	(0.000)	(0.000)	(0.000)
	sPCA(woL2)	1.000	1.000	1.000	1.000
		(0.000)	(0.000)	(0.000)	(0.000)
	oEVD	1.000	1.000	1.000	1.000
		(0.000)	(0.000)	(0.000)	(0.000)
Precision	ssDGN	0.946	0.961	**0.964**	0.964
		(0.091)	(0.076)	(0.074)	(0.074)
	ssDGNs	0.958	0.952	0.958	0.960
		(0.078)	(0.085)	(0.078)	(0.085)
	sfPCA	0.961	0.955	0.958	0.962
		(0.076)	(0.083)	(0.078)	(0.080)
	sPCA	**0.964**	0.959	0.959	0.964
		(0.074)	(0.082)	(0.082)	(0.074)
	sPCA(woL2)	0.947	**0.967**	0.961	0.956
		(0.094)	(0.067)	(0.076)	(0.087)
	oEVD	0.958	0.952	0.965	**0.965**
		(0.078)	(0.085)	(0.078)	(0.078)

**Table 3:** Simulation results: Differential gene network identification *σ*^2^ = 5.

	Methods	Scn.1	Scn.2	Scn.3	Scn.4
Accuracy	ssDGN	**0.972**	0.966	**0.980**	**0.988**
		(0.045)	(0.059)	(0.045)	(0.033)
	ssDGNs	0.970	0.960	**0.980**	0.978
		(0.046)	(0.078)	(0.040)	(0.046)
	sfPCA	**0.972**	0.954	0.976	0.984
		(0.045)	(0.071)	(0.048)	(0.037)
	sPCA	0.968	0.954	0.976	0.986
		(0.047)	(0.071)	(0.048)	(0.035)
	sPCA(woL2)	0.968	**0.970**	**0.980**	0.984
		(0.051)	(0.051)	(0.045)	(0.037)
	oEVD	**0.972**	0.954	0.978	0.984
		(0.045)	(0.071)	(0.046)	(0.037)
F.measure	ssDGN	**0.975**	**0.970**	**0.982**	**0.989**
		(0.041)	(0.051)	(0.040)	(0.030)
	ssDGNs	0.973	0.966	0.982	0.980
		(0.042)	(0.064)	(0.037)	(0.041)
	sfPCA	**0.975**	0.960	0.978	0.985
		(0.041)	(0.059)	(0.042)	(0.034)
	sPCA	0.971	0.960	0.978	0.987
		(0.043)	(0.059)	(0.042)	(0.032)
	sPCA(woL2)	0.971	0.973	**0.982**	0.985
		(0.046)	(0.045)	(0.040)	(0.034)
	oEVD	**0.975**	0.960	0.980	0.985
		(0.041)	(0.059)	(0.041)	(0.034)
TNR	ssDGN	**0.944**	0.932	**0.960**	**0.976**
		(0.091)	(0.119)	(0.090)	(0.066)
	ssDGNs	0.940	0.920	**0.960**	0.956
		(0.093)	(0.156)	(0.081)	(0.093)
	sfPCA	**0.944**	0.908	0.952	0.968
		(0.091)	(0.141)	(0.095)	(0.074)
	sPCA	0.936	0.908	0.952	0.972
		(0.094)	(0.141)	(0.095)	(0.070)
	sPCA(woL2)	0.936	**0.940**	**0.960**	0.968
		(0.103)	(0.101)	(0.090)	(0.074)
	oEVD	**0.944**	0.908	0.956	0.968
		(0.091)	(0.141)	(0.093)	(0.074)
Recall	ssDGN	1.000	1.000	1.000	1.000
		(0.000)	(0.000)	(0.000)	(0.000)
	ssDGNs	1.000	1.000	1.000	1.000
		(0.000)	(0.000)	(0.000)	(0.000)
	sfPCA	1.000	1.000	1.000	1.000
		(0.000)	(0.000)	(0.000)	(0.000)
	sPCA	1.000	1.000	1.000	1.000
		(0.000)	(0.000)	(0.000)	(0.000)
	sPCA(woL2)	1.000	1.000	1.000	1.000
		(0.000)	(0.000)	(0.000)	(0.000)
	oEVD	1.000	1.000	1.000	1.000
		(0.000)	(0.000)	(0.000)	(0.000)
Precision	ssDGN	**0.953**	**0.946**	**0.968**	**0.980**
		(0.076)	(0.091)	(0.072)	(0.055)
	ssDGNs	0.950	0.941	0.967	0.964
		(0.077)	(0.108)	(0.067)	(0.074)
	sfPCA	**0.953**	0.929	0.961	0.973
		(0.076)	(0.103)	(0.076)	(0.062)
	sPCA	0.947	0.929	0.961	0.977
		(0.079)	(0.103)	(0.076)	(0.058)
	sPCA(woL2)	0.948	0.951	0.968	0.973
		(0.082)	(0.081)	(0.072)	(0.062)
	oEVD	**0.953**	0.929	0.964	0.973
		(0.076)	(0.103)	(0.074)	(0.062)

## Gastric cancer drug resistance specific gene networks identification

We applied ssDGN to identify differentially regulated gene networks between gastric cancer drug-sensitive and -resistant cell lines based on the publicly available Sanger Genomics of Drug Sensitivity in Cancer (GDSC) dataset from the Cancer Genome Project. We considered four anti-cancer drugs, doxorubicin, mitomycin-c, 5-FU, and docetaxel, approved by the Food and Drug Administration (FDA), for gastric cancer. We used the log of IC50 values as the drug sensitivity and the expression levels of 10% of the genes with the highest variance in all cell lines, i.e., 404 genes are considered as candidate regulator and target genes. The cell lines were matched by the expression levels and the drug sensitivity, where 948, 855, 891, and 948 cell lines for doxorubicin, mitomycin-c, 5-FU, and docetaxel were matched, respectively. We then defined drug-sensitive and -resistant cell lines based on 1^*st*^ (*D*^1*st*^) and 3^*rd*^ (*D*^3*rd*^) quartiles of drug sensitivity (DS) of each drug, i.e., drug-sensitive cell lines: *DS*^*ST*^ < *D*^1*st*^ and drug-resistant cell lines: *DS*^*RS*^ > *D*^3*rd*^.

In GDSC data analysis, the linear regression model to represent gene regulatory system is given as,

$$\begin{array}{c} x_{ij}=\delta_{j}^{T}x_{i}^{\left(-j\right)}+\epsilon_{ij},\;i=1,\ldots ,n,\;j=1,\ldots ,k, \end{array}$$ (16)

where $x_{i}^{\left(-j\right)}=\left(x_{i1},\ldots ,x_{i,j-1},x_{i,j+1},\ldots ,x_{ip}\right)$, ***δ***_*j*_ = (*δ*_*j*1_, …, *δ*_*j*,*j*−1_, *δ*_*j*,*j*+1_, …, *δ*_*jp*_)^*T*^ is the regression coefficient that represents the effect of *p* candidate regulator genes ***x***_*i*_ on *j*^*th*^ target gene *x*_*ij*_ and *ϵ*_*ij*_ is a random error vector for the *j*^*th*^ target gene. For each *j*^*th*^ target gene, we consider the remaining *p* = 403 genes as candidate regulator genes. The following lasso is used to select true regulator genes and estimate the gene networks. [19],

$$\begin{array}{c} \hat{\delta }=\underset{\delta_{j}}{\text{arg}\;\text{min}}\{\sum_{i=1}^{n}{\left(x_{ij}-\delta_{j}^{T}x_{i}^{\left(-j\right)}\right)}^{2}+\lambda \sum_{k\neq j}|\delta_{k}\left|\right\}, \end{array}$$ (17)

where and λ > 0 is the regularization parameters of ***δ***_*j*_. The weight of edges ***W*** = *w*_*ij*_ is computed based on the effect of the *i*^*th*^ gene to *j*^*th*^ gene (i.e., *δ*_*ij*_) and the *j*^*th*^ gene to *i*^*th*^ gene (i.e., *δ*_*ji*_) as follows,

$$\begin{array}{c} W=w_{ij}=\frac{|\delta_{ij}\left|+\right|\delta_{ji}|}{2}. \end{array}$$ (18)

Then, the normalized Laplacian matrix ***L*** is computed by the weight of edges ***W***.

In order to identify the differentially regulated gene network, We first estimate drug sensitive and resistance -specific gene regulatory networks *NW*^*ST*^ and *NW*^*RS*^ based on *DS*^*ST*^ and *DS*^*RS*^. From the two gene networks, we extracted each 1% edges with the largest absolute value of edge size (i.e., $NW_{1\%}^{ST}$ and $NW_{1\%}^{RS}$). For the sub networks consisting of the extracted edges, we identified differentially regulated gene networks between drug-sensitive and -resistant cell lines, where only sub-networks with nodes greater than five were considered. For doxorubicin, mitomycin-c, 5-FU, and docetaxel, 13, 13, 14, and 12 sub-networks were considered for differentially regulated gene network identification. We extracted sub-networks with a p-value smaller than 0.01, and 1, 1, and 2 networks were identified for doxorubicin, 5-FU, and docetaxel, respectively. There was no differentially regulated gene network for mitomycin-c. We then matched the identified 1, 1, and 3 sub networks with $NW_{1\%}^{ST}$ and $NW_{1\%}^{RS}$, where the matched networks can be considered as drug sensitive and resistance -specific gene regulatory structures.

**Fig 2:**
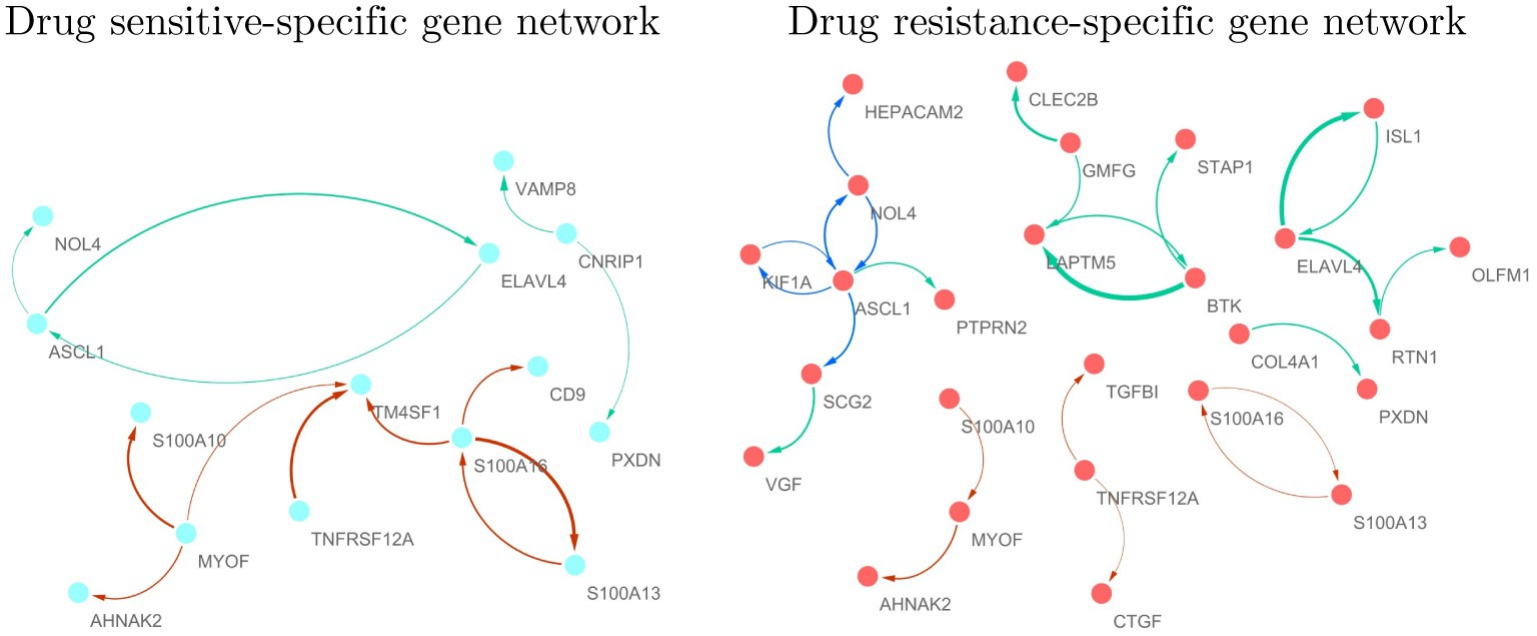
Gastric cancer drug sensitive (left) and resistance (right) -specific gene networks, where the red, blue and green indicate that the edges are extracted from 5-FU, doxorubicin and docetaxel-resistance specific gene networks, respectively.

Fig 2 shows the identified drug sensitive and resistance -specific gene networks, where color of edges indicates drugs, i.e., red: 5-FU, blue: doxorubicin, green: docetaxel. As shown in Fig 2, drug sensitive and resistance cell lines show considerably different molecular interplays, where drug resistance cell lines have relatively active and dense gene regulatory system. It implies that our strategy specify drug response -specific gene regulatory structure. We also perform drug sensitive and resistance networks estimation based on the ordinary sparse PCA (sPCA), because sPCA also show effective performance in simulation studies. The sPCA provide relatively dense networks compared with our method, i.e., 26 (13) and 100 (78) edges are identified by ssDGN and sPCA, respectively, in drug resistance (sensitive) cell lines. It can be seen that all edges identified by our method in drug resistance and sensitive cell lines are also identified by sPCA (see S1 File, tab: sPCA). Although some of edges identified by sPCA are not extracted by our method, the gene networks constructed by our approaches can be considered as crucial molecular interplays for uncovering gastric cancer drug resistance mechanism.

In order to uncover drug resistance mechanisms of gastric cancer, we focus on the identified drug resistance-specific gene networks. Table 4 shows the identified gastric cancer drug resistance markers ASCL1, NOL4, BTK, ELAVL4, LAPTM5, SCG2 that have more than three edges in the drug resistance-specific networks and their evidences, where the columns “Gastric cancer” and “Gastric cancer drugs” indicate literature related to the mechanism of gastric cancer and anti-cancer drugs uncovered by previous studies, respectively. We also perform gastric cancer resistance marker identification by using sPCA. The sPCA identify the following genes, ASCL1(13), EPS8L3(11), LGALS4(9), HSPA1A(8), NOL4(8), ELAVL4(6), IFIT3(4), SCG2(4), TRIM31(4), AGR2(3), AKR1B10(3), AKR1B10P1(3), AKR1C1(3), BTK(3), C1R(3), C1S(3), IFIT1(3), IFIT2(3), KCNMB4(3), LAPTM5(3), RTN1(3), TMSB15B(3), where the numbers in parentheses indicate the number of edges. All gastric resistance markers identified our method in Table 4 are also identified by the sPCA. It implies that the genes can be considered as crucial markers that are strongly supported by data-driven approaches.

**Table 4:** Identified gastric drug markers and evidences.

Genes	Number of edges	Gastic cancer	Chemotherapy of gastic cancer
ASCL1	8	Wang et al. [20], Nakakura et al. [21]	doxorubicin [22, 23]
NOL4	4	-	-
BTK	3	Ihler et al. [34],	docetaxel [24–26]
		Berry et al. [28]	5-FU [27]
		Zhu et al. [29]	doxorubicin [30]
ELAVL4	3	Huang et al. [31]	-
LAPTM5	3	Yang et al. [32]	docetaxel [33]
SCG2	3	Ihler et al. [34] Bae et al. [35]	Fluorouracil [36]

### Gastric cancer

ASCL1 expression levels have been considered characteristic of gastric cancer, and ASCL1 overexpression was identified as a signature of gastric tumor [21]. ASCL1 was identified as a differentially expressed gene in most gastric cancer subtypes [20]. High expression levels of SCG2 were also identified in subtype gastric cancer [34, 35]. BTK mutation was amplified in gastric cancer, which plays a critical role in anti-cancer drug resistance [28, 29]. BTK can be a therapeutic gastric cancer target since BTK expression knockdown inhibits the gastric cancer cell growth [24]. The ELAVL4 and LAPTM5 genes were also identified as differentially expressed genes in gastric cancer [31, 32]. Furthermore, SCG2 is reportedly crucial to study therapeutic strategies to treat gastric tumors [34].

### Chemotherapy of gastric cancer

Targeted ASLC1 delivery of siRNA and doxorubicin could be a potential strategy for effective neuroendocrine tumor treatment [23]. Ovarian cancer stem-like cells are highly resistant to cisplatin which is attributable to the BTK signaling overexpression [24]. CTN06, a novel BTK and ETK dual inhibitor reportedly re-sensitized cell lines to docetaxel [25]. Docetaxel substantially increased IRF3, BTK, and DDX41 expression levels [26]. BTK inhibitors reportedly synergize with 5-FU to treat drug-resistant TP53-null colon cancers, and using BTK inhibitors in combination with 5-FU is a novel therapeutic approach for colorectal cancer [27]. Additionally, doxorubicin treatment for EBNA2-positive DLBCL cells is effectively complemented with a NF-*κ*B or BTK inhibitor [30]. LAPTM5 was identified as a novel marker to enhance the resistance to docetaxel in breast cancer cells [33]. SCG2 was identified as a prognostic marker for chemotherapy in colorectal cancer by 5-FU [36].

As shown in Table 4, the drug resistance markers identified by our strategy have strong evidence as markers for gastric cancer and chemotherapy. The drug resistance-specific molecular interplays in the right side of Fig 2 may provide crucial clues to uncover the acquired drug resistance mechanisms of gastric cancer. Therefore, suppressing marker activities and their interplays can provide crucial clues to enhance the sensitivity to gastric cancer drugs.

To identify biological processes involved in the identified drug resistance (sensitive) markers, we performed gene enrichment analysis using the bioinformatics tool Database for Annotation, Visualization and Integrated Discovery (DAVID) [37]. DAVID is a web server for functional enrichment analysis and functional annotation of gene lists. GO term annotations are performed on these lists of genes with category “Molecular Function (MF)”, “Cellular Component (CC)” and “Biological Processes (BP)” based on species and databases [38]. We use the 26 (14) identified differentially regulated network in drug resistance (sensitive) cell lines as input for GO term pathway analysis.

**Fig 3:**
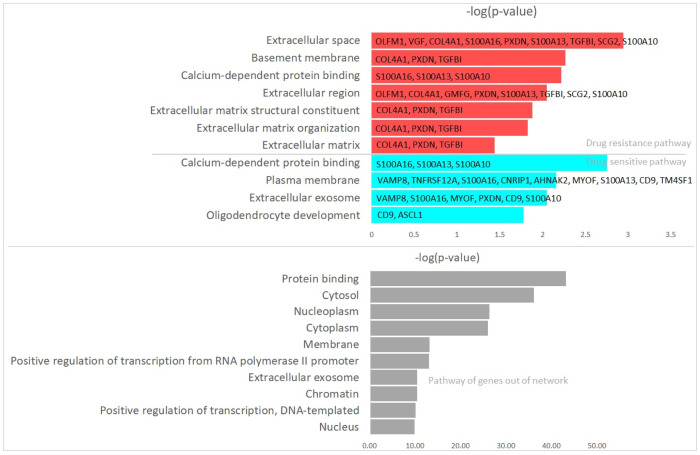
Gene Ontology (GO) term pathway analysis of gastric cancer drug resistance (sensitive) markers and genes out of network, where red and blue indicate drug resistance and sensitive -specific pathways, respectively.

Fig 3 shows the significant pathways selected with p.value < 0.05, -log(p.value) and genes comprising each pathway. As shown in Fig 3, the linked genes are enriched in a pathway, e.g., gene COL4A1 and PXDN are involved in “Extracellular matrix structural constituent”, “Extracellular matrix organization” and “Extracellular matrix” pathways. The S100A16 and S100A16 are involved in “Calcium-dependent protein binding” and “Plasma membrane” pathways. Furthermore, the genes consisting the subnetwork in bottom of drug sensitive specific cell lines, i.e., TNFRSF12A, S100A16, AHNAK2, MYOF, S100A13 and TM4SF1 are enriched in ‘Plasma membrane” pathway. The result implies that the genes linked in the networks have similar biological functions, and incorporation of the knowledge may provide biologically reliable results. GO term pathway analysis revealed that the gastric drug resistance markers are significantly enriched in the “Extracellular” associated pathway, especially the markers in “Extracellular matrix” (ECM) related pathways. The definition of the enriched “Extracellular” associated pathways is given in “THE GENE ONTOLOGY RESOURCE” (http://geneontology.org/) as follows

**Extracellular matrix**:
  A structure lying external to one or more cells providing structural support and biochemical or biomechanical cues for cells or tissues.
**Extracellular region**:
  The space external to the outer structure of a cell. This region refers to space outside the plasma membrane for cells without external protective or encapsulating structures. This term covers the host cell environment outside an intracellular parasite.
**Extracellular matrix organization**:
  A process carried out at the cellular level resulting in the assembly, arrangement of constituent parts, or disassembly of an extracellular matrix.
**Extracellular matrix structural constituent**:
  The action of a molecule that contributes to the structural integrity of the extracellular matrix.
**Extracellular space**:
  The part of a multicellular organism outside the cell, usually outside the plasma membranes and occupied by fluid.

Increased ECM stiffness has been considered a key hallmark of cancer because the ECM plays a crucial role in accelerating tumor progression, reducing the cure ratio, and promoting chemotherapy resistance of cancer cell lines [39]. The ECM can hinder chemotherapy effects by acting as a physical barrier, impeding drug access to the tumor [40]. In breast cancer cell lines, increasing matrix stiffness leads to drug resistance of cancer cell lines by impeding drug delivery ability and reducing drug sensitivity [39]. Depleted ECM components were demonstrated as essential features relating to 5-FU beneficial responses in patients with gastric cancer [41]. Enriched ECM components have been recognized as one of the primary reasons causing anti-cancer drug resistance as stiffened ECM impeded the drugs and immune cell infiltration into the tumor [41]. ECM-mediated chemoresistance is caused by a decrease in cell proliferation Keeratichamroen et al. [42], and proteins confer cell adhesion-mediated drug resistance [43]. Gene expression analysis revealed that ECM protein genes COL3A1, COL5A2, and COL15A1 are significantly upregulated in drug-resistant ovarian cancer cells [44].

We consider COL4A1, PXDN, and TGFBI key markers of the “Extracellular” associated pathways.

- COL4A1
  COL4A1 has been considered a crucial marker to drive trastuzumab resistance in gastric cancer by performing protein/gene interactions and biological process annotation analyses [45]. COL4A1 shows overexpression in gastric cancer tissues and is upregulated in trastuzumab-resistant gastric cancer cells.
- PXDN
  The high PXDN expression level has been considered a signature of drug resistance in cancer cells, and it was suggested that patients with a high PXDN expression are more likely to develop drug resistance [46].
- TGFBI
  TGFBI and its pathway are well-known drug resistance markers. The loss of TGFBI is reportedly sufficient to induce chemotherapy resistance by paclitaxel [47]. Furthermore, TGFBI knockdown leads to SKOV-3 cells being resistant to paclitaxel, and TGFBI was considered a marker for paclitaxel-resistant ovarian cancer cell line [48]. GFBI hypermethylation was reportedly considerably associated with trastuzumab resistance in HER2+ breast cancer patients [49].

These findings imply that uncovering the COL4A1, PXDN, and TGFBI mechanisms consisting of the ECM-related pathways are crucial to understanding mechanisms underlying acquired gastric cancer drug resistance. We also perform GOterm analysis for genes out of the identified networks given in Fig 2. The bottom (gray) of Fig 3 shows the most enriched 10 pathways of the genes. As shown in Fig 3, “Nucleoplasm”, “Cytosol”, and “Protein binding” are the most enriched pathways for the genes not in the drug sensitive and resistance networks. All pathways for the genes are given in S1 File. It can be seen that the genes in the network of drug sensitive and resistance cell lines are involved in the specific pathways, not in general pathway (bottom of Fig 3).

Although we did not incorporate any biomedical knowledge into the ssDGN identification, our data-driven analysis provided biologically reliable and valuable results to uncover mechanisms involved in gastric cancer drug resistance. Our GO term pathway analysis results strongly suggest that suppression and/or induction of COL4A1, PXDN and TGFBI and their molecular interplays involved in the extracellular-related pathways may provide crucial clues to enhance the chemosensitivity of gastric cancer.

Our strategy can be extended for differential gene network analysis based on various Laplacian regularizations [50–53]. We also performed gastric cancer drug resistance specific gene networks identification by using the sign adjusted quadratic Laplacian penalty $\beta_{r}^{T}L^{s}\beta_{r}$, where ***L***^*s*^ = ***S***^*T*^***LS*** with $S=\text{diag}\{\text{sgn}\left({\hat{\beta }}_{r,1}\right),\text{sgn}\left({\hat{\beta }}_{r,2},\ldots ,\text{sgn}\left({\hat{\beta }}_{r,p}\right)\right\}$. The sign adjusted quadratic Laplacian penalty was developed to incorporate the situation that neighboring genes have opposite signs of the coefficients. The method with the sign adjusted quadratic Laplacian penalty identified more subnetworks than our strategy, i.e., doxorubicin: 3 subnetworks, mitomycin-c: 2 subnetworks, 5-FU: 5 subnetworks, docetaxel: 5 subnetworks. The networks of gastric cancer drug resistance cell lines show relatively dense network (i.e., 87 edges) than its of sensitive cell lines (i.e., 58 edges) that is similar to extracted networks by the ssDGN and sPCA. The following genes are drug resistance markers identified by the method based on the sign adjusted quadratic Laplacian penalty, ASCL1 (13), EPS8L3 (11), LGALS4 (9), HSPA1A (8), NOL4 (8), ELAVL4 (6), IFIT3 (4), SCG2 (4), TRIM31 (4), AKR1B10 (3), AKR1B10P1 (3), AKR1C1 (3), BTK (3), C1R (3), C1S (3), HLA-DRB5 (3), IFIT1 (3), IFIT2 (3), LAPTM5 (3), POU2AF1 (3), RTN1 (3), TMSB15B (3), where the numbers in parentheses indicate the number of edges. The all genes identified as the gastric drug resistance markers by the ssDGN, i.e., ASCL1, NOL4, BTK, ELAVL4, LAPTM5, SCG2, are also identified by this method. All edges identified by the method based on the sign adjusted quadratic Laplacian penalty are given in the S1 File (tab: signAD).

## Discussion

In order to uncover crucial molecular interplay that plays key roles in the mechanism of gastic cancer drug resistance, we developed a novel strategy for differentially regulated gene network identification based on sparse spectral graph analysis. The proposed ssDGN estimates the eigenvalue of graph Laplacian matrices based on sparse eigen loading. Thus, we can effectively perform differential gene network identification without disturbance of noise features. The sparse eigenanalysis also leads to interpretable results; for example, we can identify crucial features to describe graph structure. In this study, we considered computational efficiency and biological interpretability and incorporated network biology knowledge into the eigenanalysis of the graph structure. Therefore, our strategy can provide biologically reliable and interpretable results.

We performed Monte Carlo simulations to illustrate the proposed strategy. The simulation results showed that the proposed ssDGN provides outstanding differential gene network identification performance. We applied our strategy to identify differentially regulated gene networks between gastric cancer drug-sensitive and -resistant cell lines. For the gastric cancer drugs, doxorubicin, Mitomycin-C, 5-FU, and Docetaxel, we estimated each drug’s sensitivity- and resistance-specific gene network and then identified the differentially regulated sub-networks between two phenotypes. The identified gastric drug resistance-specific molecular interplays and markers have strong evidence for mechanisms related to gastric cancer and its chemotherapy. The GO term pathway analysis results show that ECM-associated pathways and their members, COL4A1, PXDN, and TGFBI, are involved in gastric cancer’s acquired drug resistance mechanism. We suggest that suppression and/or induction of COL4A1, PXDN, and TGFBI, and their molecular interplays involved in the ECM-related pathways may provide crucial clues to enhance the chemosensitivity of gastric cancer.

Although the ssDGN shows effective results for differentially regulated gene network identification, our method cannot distinguish the difference between the following two networks *G*1 = {*g*1, *g*2 − *g*3} and *G*2 = {*g*1 − *g*2, *g*3} where the edge strength of g1-g2 and g2-g3 are same, because the eigenvalue of two normalized Laplacian matrices are same. This point is obvious limitation of our method. Further work remains to be done towards developing a method that can incorporate not only structure but also coordinates of network.

## Supporting information

S1 File (XLSX)

S2 File R script for Net-sEVD is available at https://drive.google.com/file/d/1UjiZ8Ex7Fv2bA3hbQz6Y9YeRFyEsnuZC/view?usp=sharing. (TXT)
